# The relationship between cardiometabolic Index and diabetic kidney disease in people with diabetes

**DOI:** 10.3389/fendo.2024.1376813

**Published:** 2025-01-09

**Authors:** Jianping Kong, Wenting Tao, Yuhong Sun, Yong Xu, Hailun Li, Jing Li

**Affiliations:** ^1^ Department of Nephrology, Nanjing Lishui People’s Hospital, Zhongda Hospital Lishui Branch, Southeast University, Nanjing, China; ^2^ Department of Critical Care Medicine, Nanjing Lishui People’s Hospital, Zhongda Hospital Lishui Branch, Southeast University, Nanjing, China; ^3^ The Department of Nursing, School of Physical Education and Health, Sichuan Institute of Industrial Technology, Deyang, China; ^4^ Department of Nephrology, The Affiliated Huai’an Hospital of Xuzhou Medical University and Huai’an Second People’s Hospital, Huai’an, China

**Keywords:** cardiometabolic index, diabetes, diabetic kidney disease, NHANES, adults

## Abstract

**Introduction:**

Studies have shown a strong correlation between the cardiometabolic index (CMI) and health issues such as diabetes, atherosclerosis, and decreased renal function. Nevertheless, the correlation between CMI and diabetic kidney disease (DKD) remains ambiguous. The objective of this study is to evaluate the correlation between CMI and DKD in patients with diabetes in the United States.

**Methods:**

The study involved individuals who were part of the National Health and Nutrition Examination Survey (NHANES) conducted between 2003 and 2018. A multivariable logistic regression analysis was employed for investigating the correlation between CMI and DKD. The study employed Generalized Additive Models (GAM) and smooth curve fitting methods for investigating the nonlinear relationship between CMI and DKD. Two-stage regression analysis was applied for investigating threshold effects in the connection between CMI and DKD. In addition, subgroup analysis and interaction tests were also carried out.

**Results:**

This analysis included a total of 6,540 adults with diabetes. After adjusting for variables including age, sex, race, education level, smoking status, household income and poverty rate, body mass index, hypertension status, aspartate aminotransferase, alanine aminotransferase, serum albumin, and serum globulin, we discovered a significant connection between CMI levels and the risk of DKD (OR=1.11, 95% CI: 1.05, 1.17, p<0.0001). Individuals with varying smoking statuses showed variations in this connection according to subgroup analysis and interaction tests (p for interaction=0.0216). Conversely, this correlation appeared similar across different genders, ages, races, BMI categories, hypertension statuses, and insulin usage among people with diabetes (all p for interaction >0.05). A nonlinear relationship existed between CMI and DKD, with threshold analysis indicating a turning point at CMI=1.7. A positive correlation was observed between CMI levels in people with diabetes and the risk of DKD when CMI exceeded 1.7.

**Conclusion:**

The risk of DKD was significantly positively correlated with the CMI levels of people with diabetes. Further larger prospective studies are required to confirm our results.

## 1 Introduction

Diabetes, as one of the most prevalent chronic metabolic diseases, has evolved into a global health challenge. In 2019, it was estimated that around 4.63 billion people, accounting for 9.3% of the population, had diagnosed or undiagnosed diabetes. The projection indicates an increase to 10.2% (about 5.78 billion) by the year 2030 and a further rise to 10.9% (around 7 billion) by 2045 (1). Long-term blood glucose rise in people with diabetes results in renal damage, both structural and functional, which is usually indicated by albuminuria and a decrease in glomerular filtration rate (2). Diabetic kidney disease (DKD) is a prevalent complication of diabetes mellitus that not only serves as a major contributor to end-stage renal disease but also significantly escalates the incidence of cardiovascular events, imposing a substantial burden on societal healthcare resources and the economy (3). Therefore, actively exploring potential risk factors for DKD is crucial for formulating effective strategies for its prevention and management.

The lipid levels in serum and the body’s obesity status are closely associated with DKD. The hyperglycemic state in people with diabetes may lead to disruptions in lipid metabolism, directly affecting the structure and function of the kidneys and thereby promoting the development of DKD (4, 5). In addition, some studies have pointed out that the change of blood lipids is an early indicator of kidney changes in diabetes mice (6). Lipids may become a new target for the treatment of DKD in the future (7). Currently, there was still a lack of validated indicators for assessing the occurrence of kidney disease in diabetic patients through lipid levels. Obesity itself is a chronic inflammatory state that can exacerbate high blood sugar and insulin resistance, similarly exerting adverse effects on the kidneys (8, 9). Some data suggested that bariatric surgery is effective in reducing the incidence of kidney disease in obese diabetic patients (10). The cardiometabolic index (CMI) is a recently developed measure linked to obesity and cholesterol levels. Its computation takes into account the waist-to-height ratio (WHtR) and the lipid ratio of triglycerides to high-density lipoprotein cholesterol (TG/HDL-C), yielding a simple and reproducible index. Studies had shown that CMI reflects an individual’s obesity and lipid levels and can be an effective predictor of diabetes (11). Furthermore, a series of studies suggested that CMI was linked to cardiovascular disease (12–15). The risk of microalbuminuria was shown to be significantly higher in subgroups with high CMI, both in the general population and in the diabetic and hypertensive populations. This suggests that CMI may be a valid predictor of the risk of kidney disease in diabetic patients. However, there is currently a lack of research focusing on the link between CMI in diabetes individuals and DKD risk.

Thus, the purpose of this research was to look at the relationship between CMI of adult diabetic patients and their odds of DKD in the US.

## 2 Subjects and methods

### 2.1 Survey description and research population

The data for this research came from the National Health and Nutrition Examination Survey (NHANES), which ran from 2003 to 2018. NHANES is a nationwide program designed, executed, and disseminated by the National Center for Health Statistics (NCHS) in the United States. Its goal is to evaluate people’s health and nutritional condition nationwide. The study implements a complex, multi-level probabilistic methodology to construct a sample reflective of the civilian noninstitutional population in America. Approval for the NHANES research protocols was obtained from the Research Ethics Review Board of the NCHS, ensuring adherence to ethical standards. Explicit written permission was obtained from everyone who participated in the poll. The public can access the detailed aspects of the study’s methodology and data-gathering techniques on the NHANES website at www.cdc.gov/nchs/nhanes/.

**Figure 1:**
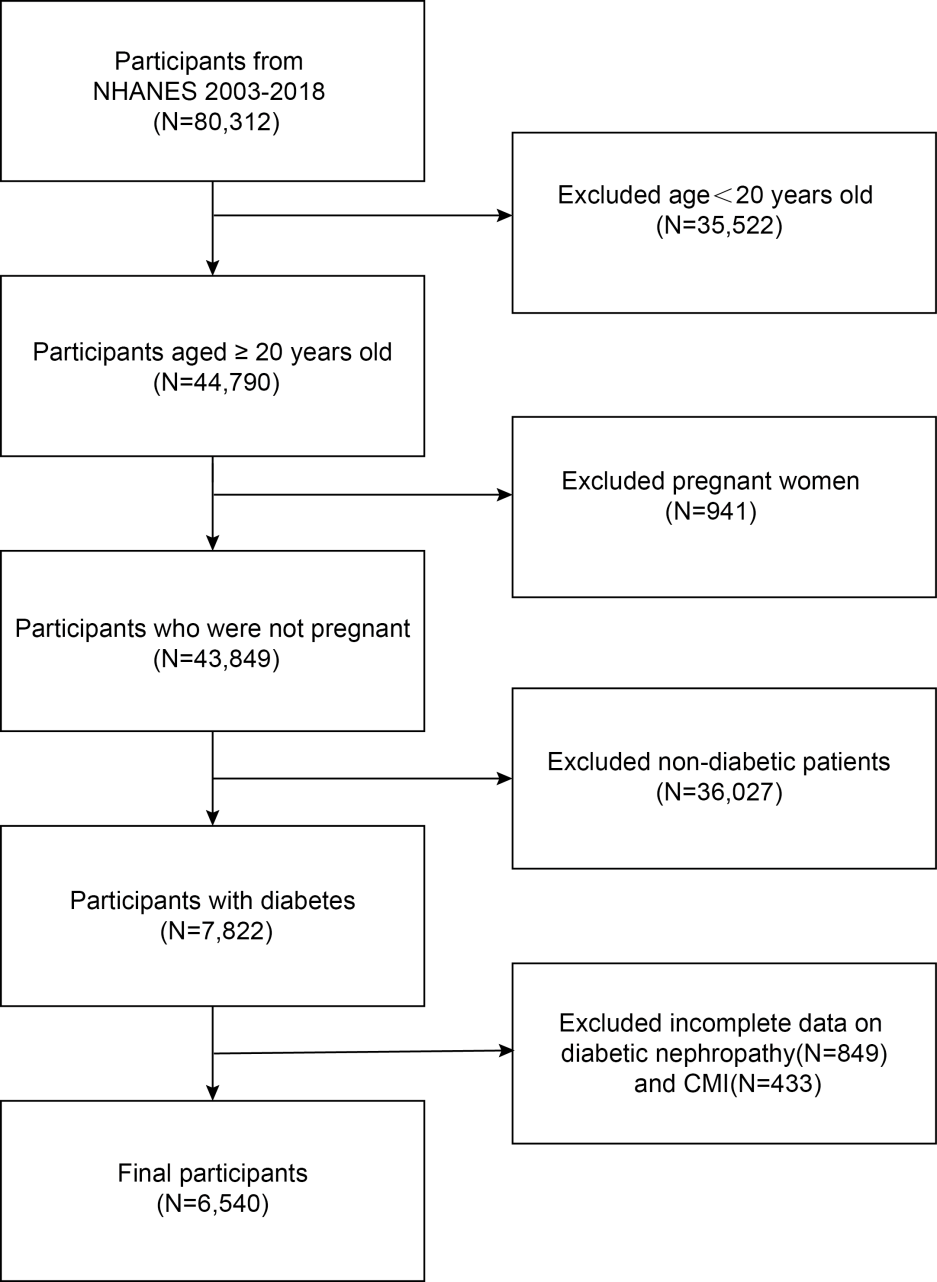
Flowchart of the sample selection.

First, we included all subjects who participated in the 2003-2018 NHANES survey and for whom data were recorded, totaling 80,312. After excluding persons under 20 years old (n=35,522), pregnant females (n=941), non-diabetic individuals (n=36,027), those with unclear DKD status (n=849), and participants with incomplete data on cardiac metabolic indices (n=433), our final analysis included 6,540 subjects (**Figure 1**).

### 2.2 Research variables

The outcome variable under investigation in this study was diabetic kidney disease (DKD). Based on the American Diabetes Correlation’s (ADA) criteria, the diagnosis of diabetes in this research was contingent upon meeting one of the following conditions: the utilization of hypoglycemic medications or a documented diagnosis by a medical professional, a hemoglobin A1c level ≥ 6.5%, fasting blood glucose ≥ 126 mg/dl, or a plasma glucose reading ≥ 200 mg/dl two hours after a meal (16). The ratio of albumin to creatinine in urine was employed to calculate the albumin-to-creatinine ratio (ACR). The estimated glomerular filtration rate (eGFR, ml/min/1.73m²) was computed utilizing the Chronic Kidney Disease Epidemiological Collaboration (CKD-EPI Cr) equation (17). In the current investigation, the diagnosis of DKD in patients was based on the presence of an eGFR < 60 mL/min/1.73m² and/or an ACR > 30 mg/g (18).

This research used CMI as its exposure variable, which is a continuous quantity. The triglyceride(TG) to high-density lipoprotein cholesterol (HDL-C) ratio and waist-to-height ratio (WHtR) were multiplied to determine CMI. Triglycerides and HDL-C levels were quantified in millimoles per liter (mmol/L), whereas height and waist circumference were evaluated in centimeters (cm) (11).

The covariates in this study included age, sex, race, smoking status, education level, body mass index (BMI), the ratio of family income to poverty (PIR), aspartate transaminase (AST), alanine transaminase (ALT), serum globulin, serum albumin, hypertension status, and insulin use. Normal weight (<25 kg/m²), overweight (25-29.9 kg/m²), and obesity (≥30 kg/m²) were the three established BMI classifications (19). The three smoking status categories were defined as follows: “never” for those who smoked less than 100 cigarettes in their lifetime, “former” for those who smoked more than 100 cigarettes but are not currently smoking, and “current” for those who have smoked more than 100 cigarettes and are presently smoking (20).

### 2.3 Statistical analysis

In this research, R (version 4.2.3) and Empowerstats (version 2.0) were used for all analyses. Continuous variables were evaluated with a weighted Student’s t-test and reported as mean ± SD. Categorical variables, presented as percentages, were assessed using a weighted chi-square test. This research used multivariate logistic regression analysis to look at the relationship between CMI and DKD. Model 1 has no adjusted variables. Model 2 adjusted for race, gender, and age. Model 3 adjusted the age, sex, race, education, smoking status, PIR, BMI, ALT, AST, hypertension, serum albumin, serum globulin. The study also utilized the generalized additive model (GAM) and smooth curve fittings to explore the nonlinear correlation between CMI and DKD. In order to investigate threshold effects in the correlation between CMI and DKD, two-stage regression analysis was used. CMI and DKD were examined in distinct groups using subgroup analysis and interaction testing. A significance level of p < 0.05 was determined.

## 3 Results

### 3.1 Baseline data

In this study, 6,540 participants with diabetes were included, with an average age of 58.98 ± 13.82 years. Of the total, 52.10% were male and 47.90% were female. There were 2,632 participants with DKD, constituting 40.24% of all participants. **Table 1** displayed the participant characteristics according to their DKD status. The findings indicated that DKD was more prevalent among older individuals, females, those with lower education levels, former smokers, and overweight individuals, as well as those with lower PIR. Additionally, the DKD group showed significantly higher rates of hypertension and insulin use compared to the non-DKD group. Furthermore, patients with DKD tended to have elevated levels of CMI, with an average of 2.86 ± 1.30.

**Table 1:** Baseline characteristics of study participants grouped by diabetes nephropathy status.

	Overall	Non-DKD	DKD	*P-value*
	N=6,540	N=3,908	N=2,632	
Age(year)	58.98 ± 13.82	56.19 ± 13.34	63.65 ± 13.33	<0.0001
Sex (%)				<0.001
Male	52.10	52.31	51.76	
Female	47.90	47.69	48.24	
Race (%)				0.3014
Mexican American	9.72	10.12	9.04	
Other Hispanic	5.78	6.01	5.39	
Non-Hispanic White	62.40	61.74	63.51	
Non-Hispanic Black	13.79	14.05	13.36	
Other Race	8.31	8.08	8.70	
Education level(%)				<0.0001
Less than high school	23.80	21.91	26.98	
High school or GED	25.21	24.28	26.77	
Above high school	50.94	53.80	46.14	
Unknown	0.05	0.02	0.11	
Smoking status(%)				<0.0001
Never	48.90	50.34	46.49	
Former	34.26	32.18	37.76	
Current	16.80	17.48	15.67	
Unknown	0.03	0.01	0.08	
BMI				0.0044
Normal weight	12.21	11.50	13.39	
Overweight	27.42	28.64	25.37	
Obese	60.37	59.86	61.24	
PIR	2.78 ± 1.61	2.89 ± 1.62	2.60 ± 1.57	<0.0001
Serum albumin (g/L)	41.48 ± 3.40	41.74 ± 3.27	41.05 ± 3.56	<0.0001
Serum globulin (g/L)	29.56 ± 4.75	29.13 ± 4.55	30.28 ± 4.99	<0.0001
ALT (U/L)	27.98 ± 27.61	28.74 ± 20.94	26.72 ± 36.10	0.0042
AST (U/L)	26.83 ± 19.66	26.85 ± 19.41	26.80 ± 20.07	0.9255
Waist Circumference (cm)	110.42 ± 16.23	109.98 ± 16.10	111.18 ± 16.42	0.0038
Height (cm)	167.58 ± 10.33	168.08 ± 10.34	166.75 ± 10.25	<0.0001
Triglyceride (mmol/L)	4.88 ± 1.25	4.91 ± 1.22	4.84 ± 1.28	0.0241
HDL (mmol/L)	1.24 ± 0.39	1.24 ± 0.36	1.24 ± 0.44	0.9588
CMI	2.82 ± 1.22	2.80 ± 1.16	2.86 ± 1.30	0.0421
Hypertension (%)	63.71	57.29	74.48	<0.0001
Injecting insulin (%)	18.48	14.52	25.13	<0.0001

Mean ± SD for continuous variables: the p-value was calculated by the weighted linear regression model. (%) for categorical variables: the p-value was calculated by the weighted chi-square test.

DKD, diabetic kidney disease; GED, general educational development; PIR, the ratio of family income to poverty; AST, aspartate transaminase; ALT, alanine transaminase; BMI, body mass index; HDL, Direct HDL-Cholesterol; CMI, cardiometabolic index.

BMI: <25 kg/m², Normal weight; 25-29.9 kg/m², Overweight; ≥30 kg/m², Obese.

### 3.2 The correlation between DKD and CMI

**Table 2** showed the correlation between CMI and DKD risk. Our research findings indicated a positive relationship between higher CMI levels and increased DKD risk in diabetic patients. However, this positive correlation varied across different models. In the model without covariate adjustment (Model 1), the prevalence of DKD in diabetes patients increased by 3% when the CMI level increased by one unit, but the correlation was not statistically relevant (OR=1.03, 95% CI: 0.99, 1.07, p=0.1924). Even when treating CMI as a categorical variable, no statistically significant correlation was observed. In other words, in Model 1, we did not find a meaningful correlation between CMI and DKD risk.

**Table 2:** The correlation between cardiometabolic index and diabetic kidney disease in patients with diabetes.

Exposure	Model 1 [OR (95% CI)], *P-*value	Model 2 [OR (95% CI)], *P-*value	Model 3 [OR (95% CI)], *P-*value
CMI (continuous)	1.03 (0.99, 1.07), 0.1924	1.17 (1.12, 1.23), <0.0001	1.11 (1.05, 1.17), <0.0001
CMI (quartile)			
Quartile 1 (≤1.9)	Reference	Reference	Reference
Quartile 2 (2.0-2.4)	0.90 (0.77, 1.04), 0.1410	0.95 (0.81, 1.10), 0.4809	0.87 (0.74, 1.03), 0.1122
Quartile 3 (2.5-3.2)	0.99 (0.86, 1.13), 0.8483	1.20 (1.04, 1.38), 0.0137	1.02 (0.88, 1.20), 0.7612
Quartile 4 (≥3.3)	1.02 (0.89, 1.17), 0.7389	1.51 (1.30, 1.75), <0.0001	1.22 (1.04, 1.44), 0.0151
*p* for trend	0.4098	<0.0001	0.0019

Model 1: no covariates were adjusted.

Model 2: age, sex, and race were adjusted.

Model 3: age, sex, race, education, smoking status, PIR, BMI, ALT, AST, hypertension, serum albumin, serum globulin were adjusted.

PIR, ratio of family income to poverty; BMI, body mass index, AST, aspartate aminotransferase; ALT, alanine aminotransferase; CMI, cardiometabolic index.

After adjusting for race, sex, and age (Model 2), a strong positive correlation emerged of CMI on DKD (OR=1.17, 95% CI: 1.12, 1.23, p<0.0001). Specifically, the prevalence of DKD increased by 17% for diabetic individuals for every unit rise in CMI. This correlation retained its statistical significance when CMI was categorized into quartiles. Diabetes patients in the third CMI quartile exhibited a 20% significant increase in DKD prevalence compared to those in the lowest CMI quartile (OR=1.20, 95% CI: 1.04, 1.38, p<0.0137), and in the highest CMI quartile of diabetes patients, the prevalence of DKD increased by 51% (OR = 1.51, 95% CI: 1.30, 1.75; p <0.0001; P for trend <0.0001). In model 2, we found that higher levels of CMI were associated with a higher prevalence of DKD.

This positive correlation continued to be statistically significant even after accounting for all variables. In Model 3, for every unit increase in CMI level, the prevalence of DKD increases by 11%. (OR=1.11, 95% CI: 1.05, 1.17, p<0.0001). Similar to Model 2, the positive correlation persisted when CMI was considered a categorical variable. In Model 3, diabetic patients in the highest CMI quartile experienced a significant 22% increase in prevalence of DKD than in those in the lowest (OR = 1.22, 95% CI: 1.04, 1.44; p=0.0151; p for trend =0.0019). In model 3, we also found that higher levels of CMI were associated with a higher prevalence of DKD.

**Figure 2:**
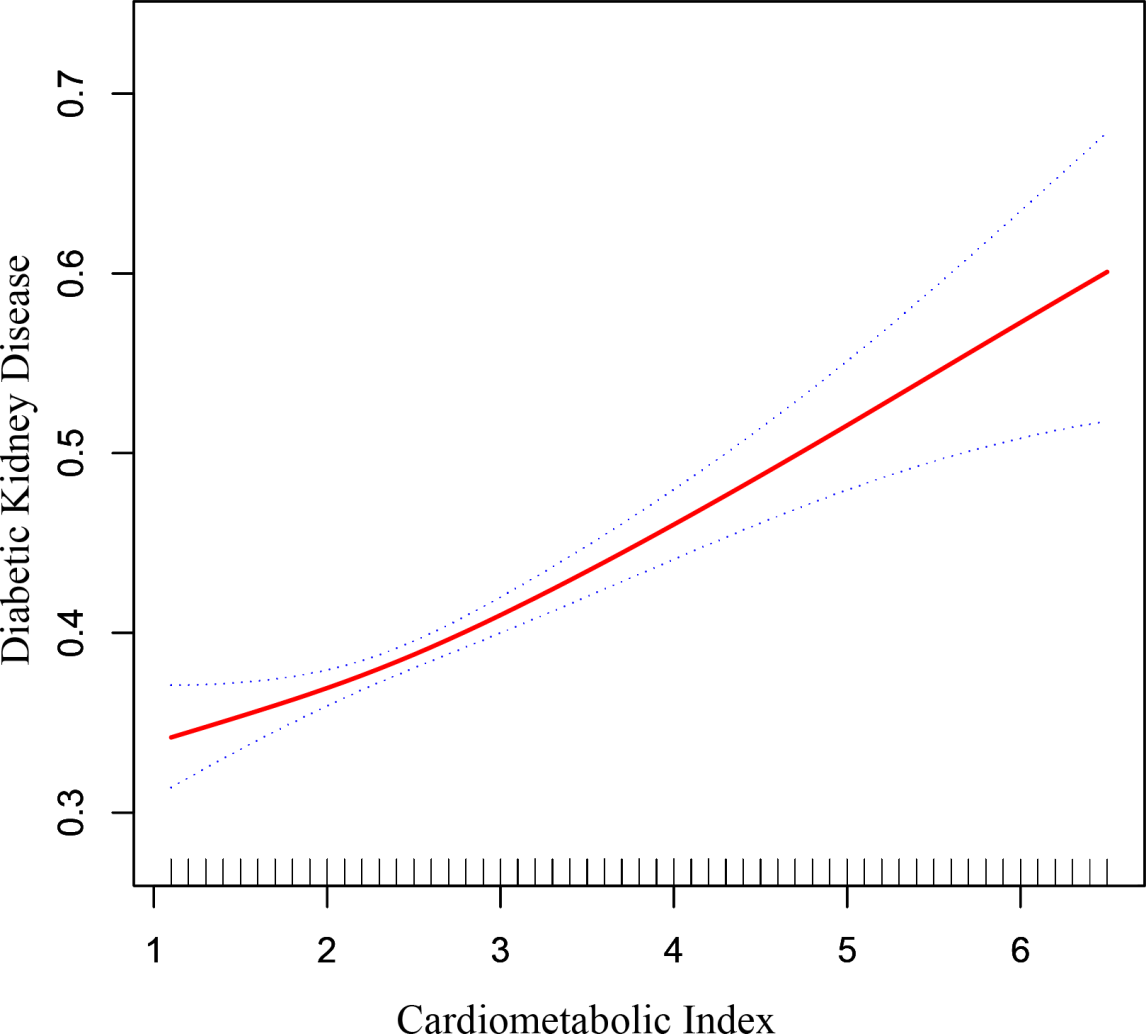
The correlation between cardiometabolic index and diabetic kidney disease in patients with diabetes. The correlation between CMI and DKD. The solid red line represents the smooth curve fit between variables. Blue bands represent the 95% of confidence interval from the fit.

**Figure 2** depicted the non-linear connection between CMI and DKD after applying smoothing curve fitting in the generalized additive model. We calculated the inflection point (K) of this relationship in the diabetic group to be 1.7 using threshold effect analysis (**Table 3**). There was no discernible relationship between the prevalence of DKD and CMI on the left side of the inflection point (OR=0.70, 95% CI: 0.46, 1.06, p=0.0919). But after CMI hit 1.7, the risk of DKD in diabetic individuals rose by 20% for every unit rise in CMI (OR=1.20, 95% CI: 1.14, 1.27, p<0.0001). This suggests that some other factors may have interfered with the effect of CMI on DKD risk before CMI reached the 1.7 level.

**Table 3:** Threshold effect analysis of cardiometabolic index on the risk of diabetic kidney disease in patients with diabetes using two-piecewise linear regression model.

Total bone mineral density	OR (95% CI), *P*-value
Fitting by the standard linear model	1.17 (1.12, 1.23), <0.0001
Fitting by the two-piecewise linear model	
Inflection point	1.7
CMI<1.7	0.70 (0.46, 1.06), 0.0919
CMI>1.7	1.20 (1.14, 1.27), <0.0001
Log likelihood ratio	0.014

The model adjusted for age, sex, race.

### 3.3 Analysis of subgroups

We conducted subgroup analyses and interaction tests to evaluate the influence of CMI on DKD risk across various diabetic groups, stratified by factors such as age, sex, race, BMI, smoking status, hypertension, and insulin usage. The findings, as shown in **Table 4**, demonstrated variable correlations. Notably, all subgroups defined by sex and hypertension status exhibited a strong positive relationship of CMI on DKD risk (all p<0.05). However, in subgroups stratified by age, race, BMI, smoking status, and insulin use, a significant positive correlation was predominantly found in patients under 60, non-Hispanic white and black individuals, other races, those overweight or obese, never or former smokers, and non-insulin users (all p<0.05). In other diabetic subgroups, this correlation was not statistically significant (all p>0.05). Interaction tests revealed a notable difference in the CMI-DKD risk correlation based on smoking status. That is, in the patients with diabetes who were not current smokers (including non-smokers and former smokers), the increase of CMI level was accompanied by the increase of DKD risk; in the current smoking diabetes patients, this relationship did not exist; this difference was statistically significant (p for interaction=0.0216). But no significant interaction effects were observed for sex, age, race, BMI, hypertension, or insulin use (all p for interaction >0.05), suggesting these factors did not significantly modify the relationship.

**Table 4:** Subgroup analysis of the correlation between cardiometabolic index and diabetic kidney disease in patients with diabetes.

	OR (95% CI), *p*-value	*p* for interaction
Stratified by age		0.1388
< 60 years old	1.12 (1.04, 1.20) 0.0033	
≥60 years old	1.03 (0.96, 1.11) 0.3850	
Stratified by sex		0.2664
Male	1.08 (1.01, 1.16) 0.0194	
Female	1.15 (1.06, 1.25) 0.0007	
Stratified by race		0.5516
Mexican American	1.06 (0.94, 1.20) 0.3554	
Other Hispanic	1.03 (0.86, 1.22) 0.7673	
Non-Hispanic White	1.14 (1.05, 1.23) 0.0015	
Non-Hispanic Black	1.14 (1.00, 1.29) 0.0430	
Other Race	1.24 (1.03, 1.50) 0.0253	
Stratified by BMI		0.9066
Normal weight	1.17 (0.95, 1.43) 0.1356	
Overweight	1.11 (0.99, 1.24) 0.0643	
Obese	1.13 (1.05, 1.21) 0.0005	
Stratified by smoking status		0.0216
Never	1.17 (1.08, 1.27) 0.0002	
Former	1.16 (1.05, 1.27) 0.0027	
Current	0.98 (0.88, 1.09) 0.6803	
Injecting insulin		0.5242
Yes	1.08 (0.95, 1.22) 0.2270	
No	1.12 (1.06, 1.19) <0.0001	
Hypertension		0.8232
Yes	1.10 (1.03, 1.18) 0.0033	
No	1.12 (1.02, 1.22) 0.0151	

In subgroup analyses stratified by age, sex, race, education, smoking status, PIR, BMI, ALT, AST, hypertension, serum albumin, serum globulin, but the model did not adjust for the stratification variables themselves.

BMI: <25 kg/m², Normal weight; 25-29.9 kg/m², Overweight; ≥30 kg/m², Obese.

## 4 Discussion

Our survey investigation, which included 6,540 adult diabetes patients, revealed a strong correlation of CMI levels on the risk of DKD. Interestingly, subgroup analysis and interaction tests indicated that this correlation varied based on smoking status, while it appeared similar across other factors such as gender, age, race, BMI categories, hypertension status, and insulin usage among diabetic patients. It is noteworthy that a nonlinear relationship existed between CMI and DKD with a turning point at CMI=1.7. More specifically, above this inflection point, CMI levels were positively correlated with an increased risk of DKD in diabetic individuals.

CMI, a recently proposed novel anthropometric index, combines blood lipid and obesity indicators to assess abnormal body fat distribution (11). Previous studies have validated the strong correlation between the levels of CMI and the risk of diabetes. Multiple logistic regression analysis showed a high positive correlation between diabetes and CMI in a Chinese research. This positive link was steady even after controlling for confounding variables including age, race, education level, income, and physical activity. ROC analysis indicated that CMI outperforms traditional BMI in distinguishing diabetes (21). Similarly, a study from Japan supported the significant positive correlation between the incidence of diabetes and CMI. The research suggested that this correlation is more pronounced in females, current smokers, individuals with sedentary lifestyles, and those with a BMI ≥ 25kg/m2. Consequently, CMI, as an indicator for assessing abnormal body fat distribution, may exhibit different predictive abilities in different populations (22). CMI’s relevance extends beyond diabetes, serving as a reliable indicator for detecting other metabolic disorders. In a retrospective cohort study involving severely obese women, CMI demonstrated better sensitivity and specificity in identifying metabolic syndrome (MetS) than traditional obesity indices such as BMI, WtHR, and body fat index (23). CMI’s effective predictive role in cardiovascular diseases has been substantiated. In a cross-sectional analysis involving 11,258 participants, Wang et al. reported that CMI is an independent determinant for left ventricular remodeling in the general rural population of China, and this impact of CMI on left ventricular remodeling is notably more significant in females (24). Furthermore, a retrospective analysis conducted by Wakabayashi and their team suggests a close correlation between CMI and the degree of carotid atherosclerosis and lower limb arterial ischemia. CMI is considered a reliable discriminative index for peripheral arterial diseases (13). Studies have proved this link between CMI and poor kidney function. In a study involving 64,313 participants, Guo et al. found that greater CMI levels are linked to a higher likelihood of chronic kidney disease (CKD), and this relationship remains stable across various age groups, obesity statuses, smoking habits, hypertension, and diabetes subgroups (25). Miao et al., in an analysis of NHANES data, identified an independent correlation between CMI and microalbuminuria, especially prominent in diabetic patients (12). In summary, the focus of previous studies had mostly been on the prediction of diabetes or decreased renal function by CMI levels. Nonetheless, it is still unclear how CMI levels and the risk of DKD in people with diabetes are related. Instead, our study focused on the correlation between CMI and its DKD risk in a diabetic population. Our results were consistent with previous studies in that in diabetic patients, elevated CMI levels were also associated with an increased risk of kidney disease.

In the medical community, studies examining the connection between blood lipid levels, obesity, and DKD have long been of interest. In a Meta-analysis incorporating 14 studies, researchers observed a significant correlation between abdominal obesity parameters (such as visceral fat area (VFA) or waist circumference (WC)) and the increased likelihood of DKD. Moreover, individuals with type 2 diabetes mellitus who suffer from DKD are more likely to exhibit abdominal obesity (26). Studies from Korea also indicated a close relationship between obesity and renal function in diabetic patients. In type 2 diabetic individuals with normal kidney function, Kim et al. discovered a strong correlation between obesity and the higher risk of renal function decrease. An independent risk factor for renal function decrease in diabetes individuals was found as a weight increase of over 10% (27). Moreover, blood lipid levels and diabetes patients’ renal function are tightly correlated, just as obesity is. Yang and colleagues found that higher levels of TG and TG/HDL-C, along with lower levels of HDL-C, were significantly correlated with the risk of DKD (28). Another study from Ethiopia revealed that type 2 diabetes patients with kidney disease exhibited significantly elevated blood lipid levels than those without renal disease (29). To sum up, higher levels of blood lipids and obesity promote the progression of kidney disease in diabetes patients, which is similar to our research results. Smoking is an independent risk factor for DKD. A study from Korea found that diagnosed diabetic patients who continue smoking after diagnosis have a significantly higher risk of DKD (30). In a Meta-analysis from China, Liao et al. discovered that, compared to never-smokers, smokers with type 2 diabetes have a greater risk of DKD (31). Surprisingly, in our study, we observed that this positive correlation was absent in diabetic patients who were currently smoking but remained stable in those who had never smoked or quit smoking. In fact, this was in line with the results of previous studies. This finding reminded us that current smoking, an important risk factor, significantly interfered with the role of CMI levels in the progression of DKD.

Despite the increasing interest among researchers in recent years regarding the correlation between CMI and diabetes as well as CKD, the precise mechanisms that underlie the correlation of CMI to DKD remain unclear. The following are several potential mechanisms. Firstly, both obesity and elevated levels of blood lipids can trigger chronic low-grade inflammation in the body, leading to the excessive release of inflammatory mediators in renal tissues. This activation of the inflammatory response promotes fibrosis in renal tissues, thereby accelerating the development of kidney disease in people with diabetes (32, 33). Moreover, high blood cholesterol levels and obesity can induce oxidative stress, leading to the overproduction of free radicals in cells. These free radicals may damage renal tissues and interact with the inflammatory process, accelerating the progression of diabetic kidney disease (34–36). Additionally, elevated blood lipid levels and obesity may promote vascular changes and the occurrence of hypertension. This may result in insufficient blood supply to the kidneys, affecting the filtration function of renal glomeruli. Studies indicate that hypertension is an independent risk factor for DKD, and when combined with lipid abnormalities and obesity, it exacerbates the risk of DKD (37–39).

Understanding the limitations of this study is crucial. Firstly, it is imperative to acknowledge that due to the cross-sectional nature of our study, we can infer correlations but not causal relationships between CMI levels and the risk of DKD in diabetic patients. Secondly, due to the lack of explicit diabetes subtyping in the NHANES data, we cannot further assess whether this correlation differs between type 1 and type 2 diabetes. Thirdly, our study involved only the U.S. population, and we were unable to clarify whether this relationship changes across regions and populations. Future research should encompass a more comprehensive investigation to validate our findings. Notwithstanding these drawbacks, our research has several advantages. Firstly, a significant strength of our study is its effectively exploration of the relationship between CMI levels and DKD risk among diabetic patients, an area previously uncharted in the literature. To provide evidence for lipid related indicators to predict the risk of diabetes nephropathy, and providing new ideas for future prevention and treatment of renal disease in diabetic patients through lipid. Secondly, our study is based on NHANES data, a nationally representative survey utilizing a complex, stratified, multistage sampling procedure, allowing our results to be applicable to the entire adult diabetic population of the country. Additionally, the large sample size provided by the nationwide NHANES data facilitates detailed subgroup analyses, enhancing our understanding of how these correlations vary across different populations. We were therefore able to analyze whether this relationship changed across age, gender, ethnicity, BMI, smoking status, hypertension and insulin use, assessing the robustness of the relationship.

## 5 Conclusion

In conclusion, our study indicated a significant positive relationship between the level of CMI in diabetic patients and the risk of DKD. This positive correlation was stable in subjects of different genders, ages, races, body mass index, hypertension, and insulin use, but varied in different smoking conditions. However, more investigation is necessary to confirm the robustness of our findings.

## Data Availability

The original contributions presented in the study are included in the article/supplementary material. Further inquiries can be directed to the corresponding author. Publicly available datasets were analyzed in this study. This data can be found here: https://www.cdc.gov/nchs/nhanes/index.htm.
